# Efficacy of immune checkpoint inhibitors according to PD‐L1 tumor proportion scores in non‐small cell lung cancer

**DOI:** 10.1111/1759-7714.13284

**Published:** 2019-12-16

**Authors:** Seongho Park, Yoo‐Duk Choi, Jieun Kim, Bo‐Gun Kho, Cheol‐Kyu Park, In‐Jae Oh, Young‐Chul Kim

**Affiliations:** ^1^ Department of Internal Medicine Chonnam National University Medical School, Chonnam National University Hwasun Hospital Hwasun South Korea; ^2^ Department of Pathology Chonnam National University Medical School, Chonnam National University Hwasun Hospital Hwasun South Korea

**Keywords:** Nivolumab, non‐small cell lung cancer, pembrolizumab, programmed cell death ligand 1

## Abstract

**Background:**

We correlated the tumor proportion score (TPS) of programmed cell death ligand 1 (PD‐L1, SP263 or 22C3) expression with the disease control rate (DCR, partial remission and stable disease), and progression free survival (PFS) after nivolumab or pembrolizumab treatment.

**Methods:**

A total of 70 case records (55 males, 15 females) of patients with non‐small cell lung cancer (NSCLC, 46 adenocarcinoma, 22 squamous cell carcinoma, and two others) were reviewed. The PD‐L1 expressions were divided into High (SP263 ≥ 30%, 22C3 ≥ 80%) and Low groups (SP263 < 30%, 22C3 < 80%). In the combined analysis, the PD‐L1 group was defined as High if either of the two stains was classified as High and defined as Low if both stains were classified as Low.

**Results:**

Among the patients treated with nivolumab (*n* = 37), the SP263 High group showed higher DCR compared to the SP263 Low group (52.6% vs. 11.1%, *P* = 0.024). In patients treated with pembrolizumab (*n* = 33), no significant difference in DCR and PFS according to PD‐L1 expression was observed. In the combined analysis (*n* = 36), patients in the PD‐L1 High group showed significantly higher DCRs than those in the PD‐L1 Low group (56.1% vs. 24.1%, *P* = 0.028). PFS was significantly longer in the PD‐L1 High group than in the Low group (medians 4.1 *vs.* 1.6 months, respectively, *P* = 0.04).

**Conclusion:**

A high expression level of PD‐L1 was correlated with a significantly higher DCR and longer PFS in NSCLC patients treated with nivolumab or pembrolizumab.

## Introduction

Lung cancer is one of the most common cancers and is a leading cause of morbidity and mortality. The crude incidence rate was reported to be 35.1 per 100 000 in Korea.^1^ About 81.8% of lung cancer is histologically classified as non‐small cell lung cancer (NSCLC),^2^, ^3^ with a five‐year survival rate of only 22.1%.^4^ The main reason for this dismal prognosis is that about 70% of the patients with NSCLC are diagnosed at stage III or IV.^2^

Because targeted therapy is potentially very effective in patients with driver mutations,^5^, ^6^ current guidelines recommend that all patients with NSCLC, especially those with adenocarcinoma, are tested for epidermal growth factor receptor (EGFR) mutations, anaplastic lymphoma kinase (ALK) gene rearrangements, ROS1, BRAF, and PD‐L1.^7^, ^8^ The first‐line treatment for patients with NSCLC without driver mutations is cytotoxic chemotherapy, immune checkpoint inhibitors or a combination of both modalities.

Treatment options are limited for patients with NSCLC whose disease progresses after first‐line cytotoxic therapy. Docetaxel had been regarded as a standard second‐line treatment for advanced NSCLC based on longer survival rates than that of the best supportive care.^9^, ^10^ Pemetrexed proved to be noninferior to docetaxel but had significantly fewer side effects.^11^

Fortunately, immune checkpoint inhibitors that work via a cancer immune escape mechanism have recently been developed and actively studied. Pembrolizumab and nivolumab are monoclonal antibodies against programmed death 1 (PD‐1) that demonstrate anti‐tumor activity in advanced NSCLC with significantly better overall survival (OS), response rate, and PFS than docetaxel in second‐line settings.^12^, ^13^, ^14^, ^15^

However, unlike existing targeted therapy, there are insufficient indicators to predict the efficacy of immune checkpoint inhibitors. Thus, the aim of this study was to correlate the expression level of programmed death ligand‐1 (PD‐L1) by immunohistochemistry (IHC) in tissue specimens with survival and the response rate to PD‐1 inhibitors.

## Methods

The medical records of 70 consecutive patients who received nivolumab or pembrolizumab in the author's institution from September 2016 until February 2018 were retrospectively analyzed. Histologically, 46 patients had adenocarcinomas, 22 had squamous cell carcinoma, and two had NSCLC‐NOS (not otherwise specified). A total of 37 patients were treated with nivolumab and 33 with pembrolizumab (Table 1). Less than half of the patients (*n* = 33) received immune checkpoint inhibitors as second‐line treatment and the rest (*n* = 37) of the patients were treated with later‐line therapy (3rd–8th line). There was no statistically significant difference in the baseline clinical characteristics between the two groups.

**Table 1:** Characteristics of patients treated with nivolumab or pembrolizumab

	Nivolumab *n* = 37	Pembrolizumab *n* = 33	*P*‐value
Sex (M/F)	29/8	26/7	1.00
Age (mean ± SD)	67.2 ± 9.3	65.1 ± 9.3	0.350
Weight (kg, mean ± SD)	58.7 ± 10.8	64.2 ± 11.0	0.039
Height (cm, mean ± SD)	163.5 ± 7.9	164.6 ± 6.1	0.515
Smoke (Yes/No)	29/8	25/8	0.60
SQC/ADC/LCC	12/24/1	10/22/1	0.980
Line of treatment (2/3/4/5/6/7/8)	18/6/7/3/2/0/1	15/7/6/4/0/1/0	0.629
EGFR (mutant/wild/NT)	5/22/10	6/20/7	0.783
ALK (mutant/wild/NT)	2/20/15	3/19/11	0.735
EGFR or ALK (mutant/wild)	7/17	9/15	0.759
SP263 (≥30/<30%/NT)	19/18/0	12/5/16	0.302
22C3 (≥80/<80%/NT)	2/17/18	18/15/0	0.004
PD‐L1 (High/Low)†	19/12	22/4	0.098
Cycles (mean ± SD)	4.9 ± 4.4	5.1 ± 3.5	0.863
Response (PR/SD/PD/NE)	4/8/23/2	6/12/13/2	0.407
Response rate (PR)	10.8%	18.2%	0.288
Disease control rate (PR + SD)	32.4%	54.5%	0.152

ADC, adenocarcinoma; ALK, anaplastic lymphoma kinase; EGFR, epidermal growth factor receptor; LCC, large cell carcinoma; NE, not evaluable; NT, not tested; PD, progressive disease; PR, partial remission; SD, stable disease; SD, standard deviation; SQC, squamous cell carcinoma.

† In the combined analysis, the PD‐L1 group was defined as High if either of the two stains was classified as High, and defined as Low if both stains were classified as Low.

PD‐L1 expression was assessed by two immunohistochemical stains, SP263^16^ and 22C3,^17^ and the results were recorded as tumor proportion scores (TPSs). In‐house immunohistochemistry using the Ventana platform was used for the SP263 assay (Roche). For the 22C3 assay, unstained slides were sent to the Seoul Clinical Laboratory (Gyeonggi‐do, Korea), where the samples were tested using the PharmDx DACO platform. Both tests were interpreted with criteria requiring that viable tumor cells with TPS ≥50% exhibit membrane staining of any intensity. The PD‐L1 expressions were divided into High or Low groups according to the median TPS values. In the combined analysis, patients in the PD‐L1 group were defined as High if either of the two stains were classified as High and defined as Low if both stains were grouped as Low.

According to the Korean Health Insurance Review and Assessment (KHIRA) reimbursement guidelines, pembrolizumab can be used for patients with ≥50% expression of PD‐L1 using 22C3 immunohistochemistry and nivolumab for patients with ≥10% PD‐L1 using SP263 immunohistochemistry. Since the majority of the TPS in the study population were higher than those of the representative clinical trials,^12^, ^13^, ^14^, ^15^ the same cutoff points could not be used.

Treatment efficacy was evaluated according to the response evaluation criteria in solid tumors (RECIST version 1.1)^18^ and the responses were classified into three groups: partial remission (*n* = 10), disease control (defined as partial remission and stable disease, *n* = 30), progressive disease (*n* = 36), and not evaluable (*n* = 4).

PFS was defined as the time at which the disease progressed or the patient died based on the time of administration of immune checkpoint inhibitors and was analyzed using the Kaplan‐Meier method. Since this report was a retrospective observational study, disease progression was recorded at the discretion of the physician according to the radiologic findings. Thus, the confirmation of disease progression was not performed for every patient. OS was defined as the time at which the patient died based on the time of administration of inhibitors.

Statistical significance was assessed using the chi‐squared test, Student's paired *t*‐tests, the log‐rank test, and the Cox proportional hazard model. Statistical analyses were performed using R statistics^19^ and *P*‐values less than 0.05 were considered statistically significant. This study was approved by the Institutional Review Board of the author's institution (CNUHH‐2019‐197).

## Results

### PD‐L1 expression

**Figure 1:**
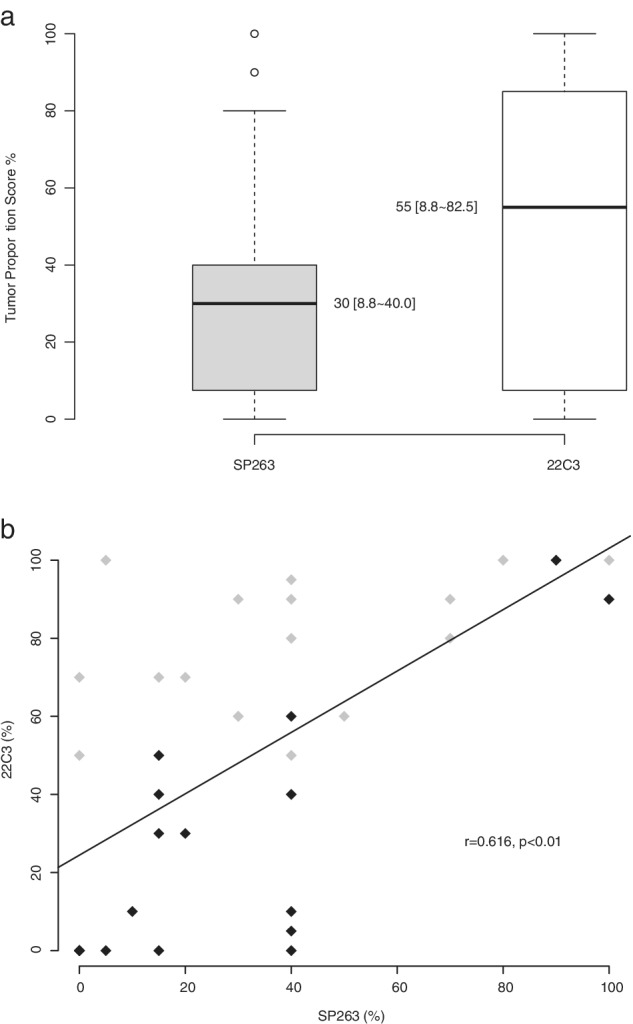
Comparison (**a**) and correlation (**b**) of PD‐L1 (SP263 and 22C3) expression in 36 patients tested with both antibodies. The data are presented as median and interquartile range. TPS, tumor proportion score. 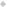 Pembrolizumab; 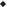 Nivolumab.

The median value of SP263 in patients treated with nivolumab was 30% (standard error, SE: 5.4%) and that of 22C3 in patients who received pembrolizumab was 80% (SE: 3.2%). Thus, PD‐L1 expression was divided into High (SP263 ≥ 30%, 22C3 ≥ 80%) and Low groups (SP263 < 30%, 22C3 < 80%). Both SP263 and 22C3 were evaluated in 36 patients and the TPS of both stains showed a significant positive correlation (Poisson R = 0.617, *P* < 0.01, Fig 1). However, the TPS of 22C3 was significantly higher than that of SP263 (mean difference 17.6%, SD 5.0%, Student's paired *t*‐test, *P* = 0.001, Fig 1).

### Overall response rate (ORR) and disease control rate (DCR)

**Figure 2:**
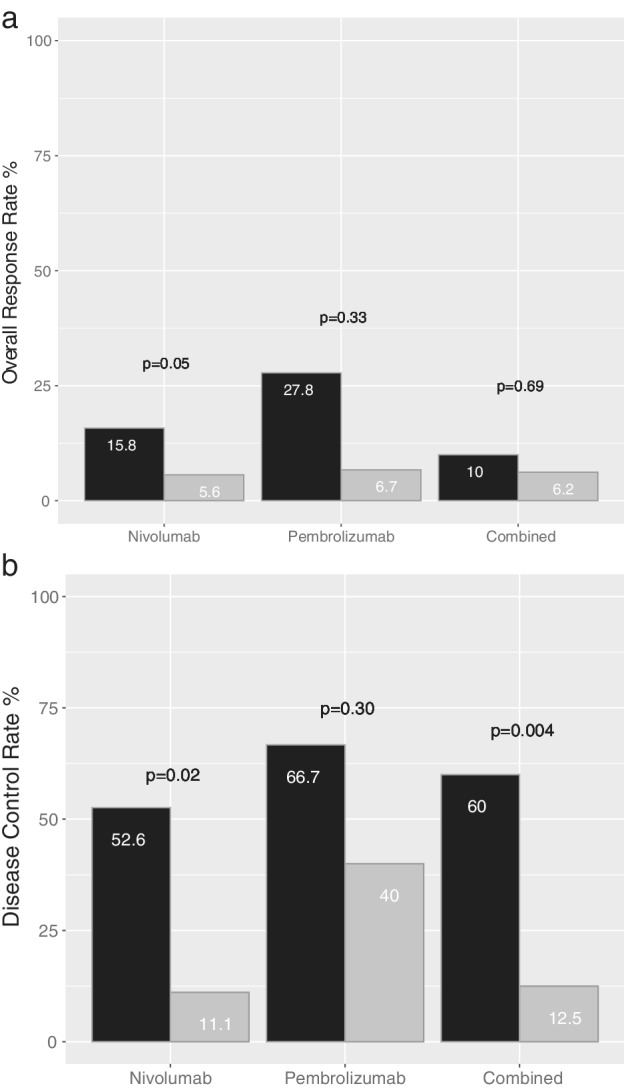
The overall response rate (**a**) and disease control rate (**b**) of PD‐L1 High (black) and Low (grey) groups of patients treated with nivolumab (*n* = 37), pembrolizumab (*n* = 33), and the combination (*n* = 36). 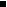 High, 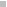 Low.

The ORR was 14.3% in 70 patients and numerically higher in the pembrolizumab group (18.2%) compared to the nivolumab group (10.8%, Table 1). There was no significant difference in the ORR according to PD‐L1 expression (Fig 2a).

The DCR was also numerically higher in the pembrolizumab group (54.5%) compared to the nivolumab group (32.4%, Table 1). DCRs were compared with PD‐L1 expression (Fig 2b). In the nivolumab group (*n* = 37), the SP263 High‐expression group showed higher DCRs compared to the Low‐expression group (52.6% vs. 11.1%, respectively, *P* = 0.024). In patients treated with pembrolizumab (*n* = 33), the DCR was numerically higher in the 22C3 High‐expression group compared to the Low‐expression group (66.7% vs. 40.0%, respectively, *P* = 0.295).

We also performed an integrated analysis comparing the response rates using 36 cases where TPS was measured using both antibodies. Although there was no difference in the ORR, significantly higher DCRs were observed in the PD‐L1 High group (60.0%) compared to the PD‐L1 Low group (12.5%, *P* = 0.004).

### Progression‐free and overall survival

**Figure 3:**
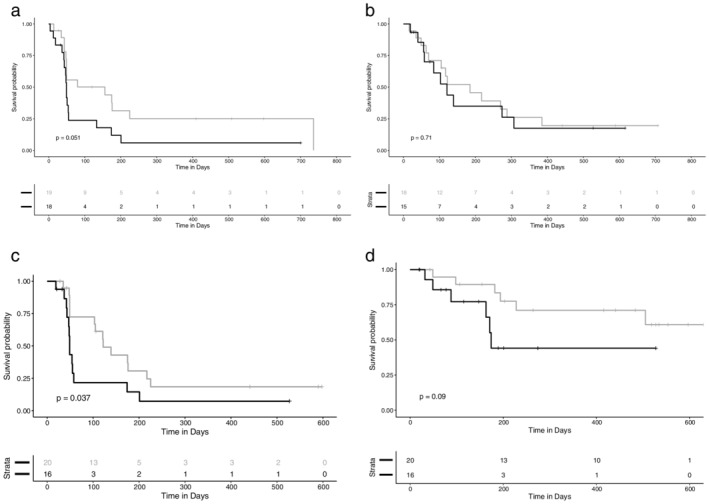
Progression‐free survival in PD‐L1 High and Low groups of patients treated with nivolumab (**a**, *n* = 37), pembrolizumab (**b**, *n* = 33), the combination (**c**, *n* = 36), and overall survival (**d**, *n* = 36), strata 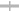 PD‐L_1_=High 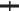
PD‐L_1_=Low.

Within the median PFS follow‐up duration of 19.6 months (589 days, 95% confidence interval [CI]: 441–not calculated), events occurred in 53 patients (75.7% maturity). The median PFS of 70 patients was calculated as 103 days (3.4 months, 44–75 days). PFS was compared with the PD‐L1 expression levels in patients treated with nivolumab (A), pembrolizumab (B), or the combination (C) (Fig 3). In the case of nivolumab (*n* = 37), the SP263 High‐expression group showed numerically longer PFS compared to the Low‐expression group (*P* = 0.05). In the case of pembrolizumab, there was no significant difference in PFS between the 22C3 High and Low‐expression groups (*P* = 0.71). However, in the combined analysis (*n* = 36), patients in the PD‐L1 High group showed significantly longer PFS than the PD‐L1 Low group (median 122 vs. 49 days, respectively, *P* = 0.037). In univariate analysis using the Cox proportional hazard model, no significant variable except PD‐L1 TPS was noted (Table 2).

**Table 2:** Univariate analysis predicting progression‐free survival

Variable	Hazard Ratio	95% confidence interval	*P*‐value
PD‐L1 (Low, high)	0.46	0.21–0.98	0.04
Age (<66, ≥66)	0.90	0.52–1.55	0.70
Sex (F, M)	1.10	0.56–2.15	0.79
Smoking (Never, ever smoker)	1.08	0.56–2.12	0.82
EGFR or ALK (Wild, mutant)	1.10	0.56–2.19	0.78
Histology (Adenocarcinoma, others)	1.05	0.59–1.87	0.88

EGFR, epidermal growth factor receptor; ALK, anaplastic lymphoma kinase genes.

Within the median OS follow‐up duration of 15.9 months (476 days, 95% CI: 274–531), events occurred in 27 patients (38.6% maturity). The median OS was 524 days (17.5 months, 318—not calculated). In a combined analysis (*n* = 36), a trend toward longer OS in the PD‐L1 High group was observed (Fig 3d).

## Discussion

Indications for the use of immune checkpoints inhibitors in patients with NSCLC have been expanding. They are efficacious, not only as second‐line treatment^12^, ^13^, ^14^ but also as first‐line therapy, alone or in combination with cytotoxic chemotherapy. When used as a first‐line treatment in advanced NSCLC with 1% or more PD‐L1 expression, nivolumab did not increase PFS compared to conventional chemotherapy but demonstrated stability and noninferiority.^20^ However, pembrolizumab was associated with significantly longer PFS and OS than platinum‐based chemotherapy when used in patients with advanced NSCLC and PD‐L1 expression in at least 50% of the tumor cells.^15^ Also, patients given pembrolizumab were shown to have significantly longer PFS and OS when used in combination with conventional chemotherapy in metastatic NSCLC, regardless of PD‐L1 expression.^21^

Tumor cell death is triggered by activated T‐lymphocytes. However, cancer cells are able to proliferate because they have mechanisms to avoid this immune process. One such process is the immune checkpoint pathway. Nivolumab and pembrolizumab are representative drugs that inhibit this process. They are classified as PD‐1 inhibitors and act on the interaction between PD‐L1 expressed on tumor cells and PD‐1 expressed on activated T‐cells, thereby blocking the inhibition of T‐cells and inducing tumor death.^22^ Therefore, many studies have suggested the possibility of using PD‐L1 expression levels as a predictor of the response to immune checkpoint inhibitors.^23^, ^24^

Previous studies have shown that immune checkpoint inhibitors have various effects depending on the PD‐L1 expression status of the patient. Using NSCLC with a PD‐L1 TPS of 50% or more, pembrolizumab showed significant benefit compared to chemotherapy as a second‐ and first‐line treatment. Nivolumab was superior to chemotherapy for NSCLC as a second‐line treatment, regardless of the degree of PD‐L1 expression,^12^, ^13^ but it was not significantly better than chemotherapy as a first‐line treatment.^20^ In other words, immunohistochemical staining of PD‐L1 expression using monoclonal antibodies is not a perfect predictor of treatment efficacy.

However, considering the mechanism of action of the drug and the results of this study, PD‐L1 expression can be used as a predictive marker of efficacy, especially for nivolumab. Since we used nivolumab for patients with ≥10% PD‐L1 expression and pembrolizumab for those with ≥50% expression according to the Korean Health Insurance Review and Assessment (KHIRA) reimbursement guidelines, the range of PD‐L1 expression was wider for patients treated with nivolumab than for those treated with pembrolizumab. This may explain the greater difference in efficacy in patients treated with nivolumab compared to pembrolizumab according to PD‐L1 expression in this study.

Alternatively, immune checkpoint inhibitors generally have a better efficacy when the degree of tumor mutation burden (TMB) is higher. Specifically, it has been reported that TMB may be a better predictor of the efficacy of nivolumab than PD‐L1 expression.^25^, ^26^ Other than PD‐L1 expression and TMB, the T‐cell inflamed gene expression profile may be used as a marker of immunotherapy in the future.^27^

In this study, we also observed a significant correlation between PD‐L1 immunohistochemical staining for SP263 and 22C3. This is consistent with the results of a blueprint project conducted by the International Association for the Study of Lung Cancer.^28^ However, we observed a significant difference in TPS between the two scoring platforms. Therefore, differences between the TPS staining methods should be considered.

This study had two limitations; sampling bias and the size of the population. Since we used nivolumab for patients with PD‐L1 TPS ≥10% and pembrolizumab for those with PD‐L1 TPS ≥50% according to the KHIRA reimbursement guidelines, the efficacy and survival of patients in the pembrolizumab group were likely to be better than those in the nivolumab group. As the TPS of the majority of the study population in the pembrolizumab group were higher than those in the representative clinical trials, we could not use the cutoff points used in previous trials. Another problem was the small sample size of this study which was too small to make comparisons with reasonable statistical power. Since we did not intend to propose certain cutoff values for PD‐L1 TPS, we just compared the differences in efficacy according to high versus low TPS.

However, with this small retrospective study, we observed that the high expression of PD‐L1 correlated with significantly higher DCRs and longer PFS in NSCLC patients treated with nivolumab or pembrolizumab. Although a correlation was observed between immunohistochemical stains with different monoclonal antibodies, we should consider significant differences in TPS in different staining platforms.

## Disclosure

The authors confirm there are no conflicting interests.
